# Multiple Organ‐Dysfunction Secondary to Multiple Bee Sting: A Case Report From Tanzania

**DOI:** 10.1002/ccr3.73392

**Published:** 2026-09-01

**Authors:** Kelvin M. Musa, Auson Albert, Jane G. Zakaria, Ronald G. Mbwasi, Anorld David, Elton Meleki

**Affiliations:** ^1^ Department of Paediatrics and Child Health Kilimanjaro Christian Medical Centre Moshi Tanzania; ^2^ Kilimanjaro Christian Medical University College Moshi Tanzania

**Keywords:** anaphylaxis, bee sting, children, envenomation, haemolysis, multisystem toxicity, rhabdomyolysis, Tanzania

## Abstract

An 8‐year‐old boy sustained over 200 bee stings, developing airway‐threatening facial oedema requiring intubation and ICU admission, with severe anemia (Hb 3.2 g/dL), rhabdomyolysis, intravascular haemolysis and marked hepatic injury (AST 2505 U/L), yet preserved renal function. He recovered without dialysis, extubated by Day 3 and discharged by Day 8.

## Key Clinical Message

An 8‐year‐old boy survived over 200 bee stings with airway‐threatening anaphylaxis and full toxic envenomation syndrome haemolysis, rhabdomyolysis, hepatic injury, coagulopathy yet avoided acute kidney injury, unlike most regional reports. Early intubation and aggressive fluid resuscitation before pigment nephropathy developed enabled full recovery without antivenom or dialysis.

## 1 Introduction

Bee stings are common in many parts of the world, particularly in tropical and rural areas where people live and work close to hives [1]. Most stings cause only a local reaction with pain and swelling that settles without treatment [2]. The outcome depends largely on the number of stings, the body size of the person stung, and whether they have been sensitized to venom before.

Three clinical patterns are recognized. A small number of stings usually produces a local reaction. In a sensitized person, even a single sting may trigger an IgE‐mediated systemic allergic reaction, including anaphylaxis. When a person receives many stings at once, the cumulative venom burden may produce a toxic envenomation syndrome affecting several organ systems [3, 4].

Massive envenomation is generally reported at a threshold of about 50 stings or more. The burden required to produce systemic toxicity varies considerably between individuals and is influenced by body weight, with severe toxicity described at approximately 1 sting/kg and fatal outcomes after several hundred stings in adults [5].

Bee venom contains vasoactive amines, enzymes and peptides. Melittin, which makes up a large proportion of venom by dry weight, is cytotoxic and nephrotoxic, while phospholipase A2 contributes to cell membrane injury and haemolysis [6, 7].

The clinical course may be biphasic. An early allergic reaction may occur within minutes to hours of the stings. This may then be followed, over the next 24 to 72 h, by delayed systemic toxicity that includes intravascular haemolysis, rhabdomyolysis, hepatic injury, thrombocytopenia, coagulopathy and acute kidney injury [8].

Acute kidney injury is among the most frequently reported severe complications. Its development is multifactorial, arising from pigment nephropathy due to hemoglobin and myoglobin, hypovolemia and shock, direct venom‐mediated tubular cytotoxicity, and the haemodynamic effects of anaphylaxis itself [4, 5, 9]. No antivenom exists for bee envenomation, so treatment rests on early recognition and aggressive supportive care.

In sub‐Saharan Africa the African honeybee ( *Apis mellifera scutellata* ) is widespread and known for aggressive mass attacks, yet pediatric reports remain few [9, 10]. We report an unusual case of a 8 years old male patient who completely recovered from life‐threatening multisystem toxicity following more than 200 bee stings without renal compromise, illustrating that early aggressive supportive care can prevent pigment nephropathy even when antivenom and dialysis are unavailable.

## 2 Case Presentation

### 2.1 Patient Demographics and History

An 8‐year‐old boy weighing 22 kg presented to our emergency department with difficulty in breathing and facial swelling of 24 h' duration. The symptoms began after he was attacked by a swarm of bees while playing outdoors, he was rushed to the district hospital and arrived after 1 h and stayed for more than 13 h before referred to our setting for father management.

He had sustained more than 200 stings, predominantly involving the head, face and upper body. This represented a venom burden well above the approximate 1 sting/kg of body weight threshold associated with systemic toxicity [11]. There was no history of loss of consciousness, convulsions, fever, vomiting or diarrhea. He had no known chronic illness and no previous history of allergy.

### 2.2 Examination Findings

On arrival he was in severe respiratory distress, with a respiratory rate of 56 breaths/min and increased work of breathing. He was severely pale. His voice was hoarse, and coarse sounds transmitted from the upper airway were heard on auscultation of the chest, with bilateral air entry.

**FIGURE 1:**
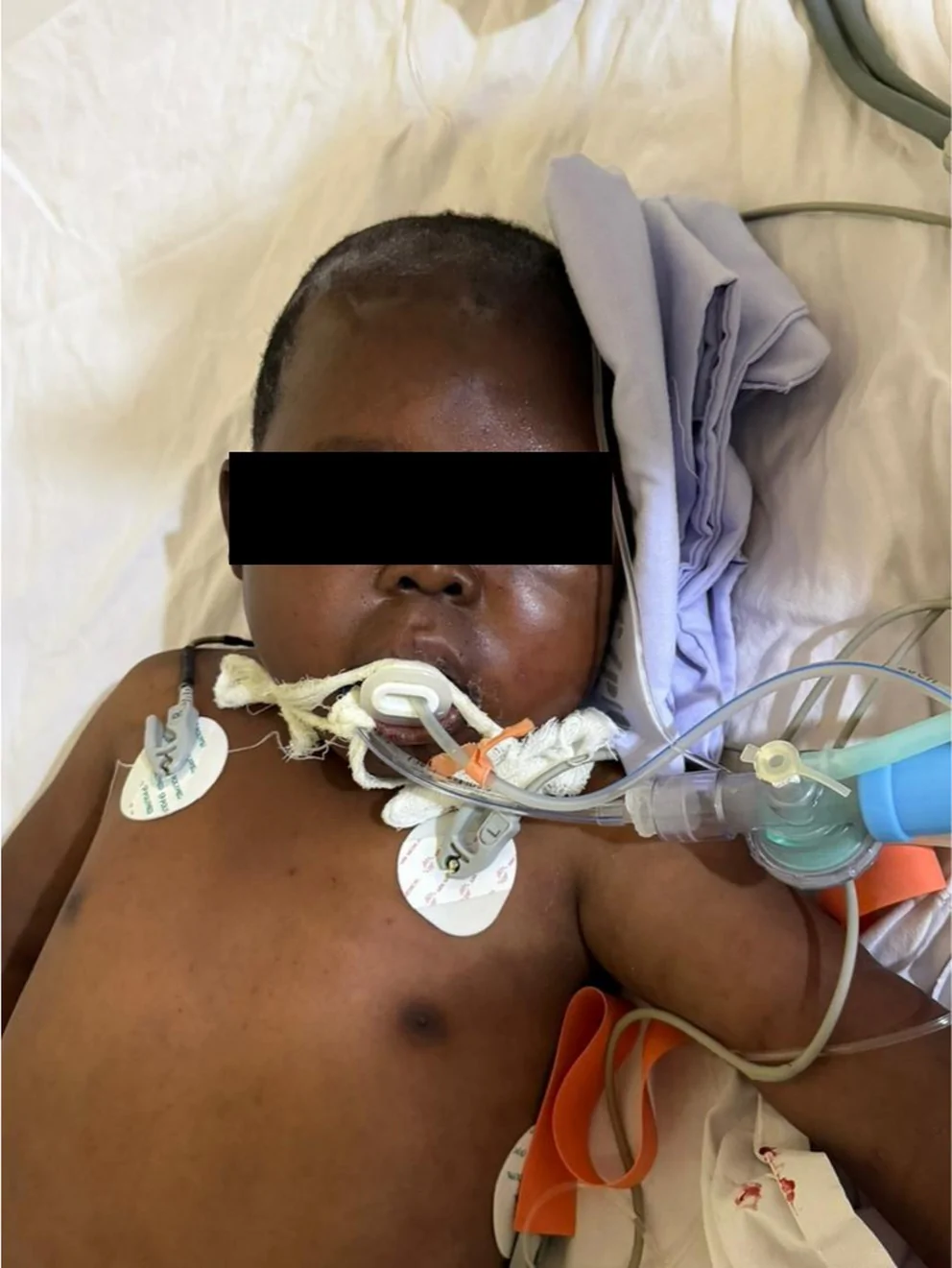
Clinical photograph of the patient demonstrating severe facial and periorbital oedema following massive bee envenomation. The patient is intubated and mechanically ventilated via an orotracheal tube. The ocular region has been masked for de‐identification. Written informed consent for publication was obtained from the parent/legal guardian.

There was marked oedema of the face and periorbital region extending to the upper arms, with numerous sting sites over the scalp, face and upper trunk (Figure 1). He was not jaundiced and had no lower limb oedema.

He was tachycardic at 138 beats/min and normotensive, with a blood pressure of 107/70 mmHg which was below 90th percentile, warm peripheries and a capillary refill time of less than 2 s. He was alert with a Glasgow Coma Scale of 15/15, equal and reactive pupils and no focal neurological deficit. Random blood glucose was 10 mmol/L and had estimated urine out of 1.2 mL/kg/h. The abdomen was soft and non‐tender with no organomegaly, and bowel sounds were normal.

### 2.3 Clinical Impression and Emergency Management

A clinical diagnosis of severe envenomation following massive bee stings, with anaphylaxis and progressive upper airway swelling, was made.

Because the facial and upper airway swelling was worsening and he was in severe respiratory distress, emergency endotracheal intubation was performed using a 5.0 mm endotracheal tube. Marked oedema of the oropharynx was seen at laryngoscopy.

He received intramuscular adrenaline 0.2 mg, intravenous dexamethasone, nebulised adrenaline, intravenous fluids as maintenance at rate of 62 mL/h, intravenous ceftriaxone and intravenous paracetamol. A urinary catheter was inserted and he was transferred to the pediatric intensive care unit.

### 2.4 Intensive Care Management

In the intensive care unit he was maintained on mechanical ventilation with sedation. He was transfused two units (450 mL) of packed red cells for the severe anemia.

Intravenous fluids were given at above‐maintenance rates with close monitoring of urine output, in order to reduce the risk of pigment‐induced kidney injury. Nebulised adrenaline was continued for the airway oedema and normal maintanance of adrenaline at a rate 0.05mcg/Kg/min, and the coagulopathy was managed supportively. No antivenom was available or given, and renal replacement therapy was not required.

### 2.5 Investigations

The full blood picture showed severe anemia with a hemoglobin of 3.2 g/dL. The red cell indices showed a microcytic hypochromic picture, with a mean corpuscular volume of 59.3 fL and a mean corpuscular hemoglobin of 15.8 pg., and the red cell distribution width was raised at 23.7%.

There was leucocytosis of 37.17 × 10^9^/L with neutrophil predominance (34.6 × 10^9^/L, 93%). The platelet count was raised at 649 × 10^9^/L.

The coagulation profile showed a prolonged prothrombin time of 22.0 s with an INR of 2.19. Liver enzymes were markedly elevated, with an AST of 2505 U/L and an ALT of 411.8 U/L, and the albumin was 34.9 g/L with a normal total bilirubin. D‐dimer and Fibrin degradation products (FDP) were not done due to financial constraints.

Creatine kinase was elevated at 1173 IU/L and lactate dehydrogenase at 1519 U/L, and urine myoglobin was positive. These findings were suggestive of rhabdomyolysis with intravascular haemolysis.

Serum sodium, chloride, and urea were normal, and potassium was mildly raised at 5.29 mmol/L. Renal function was normal at presentation and remained normal throughout the admission, with a serum creatinine of 34 μmol/L on admission and 33 μmol/L thereafter. C‐reactive protein was raised at 150.7 mg/L.

On repeat testing the hemoglobin rose to 9.1 g/dL following transfusion. The white cell count fell to 12.5 × 10^9^/L, the platelet count settled to 321 × 10^9^/L and the INR corrected to 1.10. The transaminases declined progressively, with AST falling from 2505 to 892 and then to 56.9 U/L, and ALT from 411.8 to 635.2 and then to 275.9 U/L. The serial laboratory results are summarized in Table 1.

**TABLE 1:** Laboratory results during admission.

Parameter (unit)	Reference	Day 1 (admission)	Day 3	Day 6
Hemoglobin (g/dL)	11.0–15.0	3.2 (L)	9.1*	—
MCV (fL)	80–100	59.3 (L)	75.9 (L)	—
MCH (pg)	27–32	15.8 (L)	25.0 (L)	—
RDW (%)	11–16	23.7 (H)	26.1 (H)	—
Leucocytes (×10^9^/L)	4.0–11.0	37.17 (H)	12.50 (H)	—
Neutrophils (×10^9^/L)	2.0–6.9	34.6 (H)	10.8 (H)	—
Platelets (×10^9^/L)	150–500	649 (H)	321	—
Prothrombin time (s)	9.4–12.0	22.0 (H)	11.6	—
INR	—	2.19	1.10	—
AST (U/L)	2–40	2505 (H)	892 (H)	56.9 (H)
ALT (U/L)	2–41	411.8 (H)	635.2 (H)	275.9 (H)
Total bilirubin (μmol/L)	0–21	—	6.9	—
Albumin (g/L)	35–52	—	34.9 (L)	—
Sodium (mmol/L)	136–145	137.2	—	—
Potassium (mmol/L)	3.5–5.1	5.29 (H)	—	—
Urea (mmol/L)	0–8.3	5.5	5.1	—
Creatinine (μmol/L)	62–106	34	33	—
Creatine kinase (IU/L)	38–174	—	—	1173 (H)
Lactate dehydrogenase (U/L)	240–480	—	—	1519 (H)
Urine myoglobin	Negative	Positive	—	—
C‐reactive protein (mg/L)	0–3	—	—	150.7 (H)

Abbreviations: “—”, not measured on that day; H, high; L, low.

* Hemoglobin on day 3 was measured after transfusion of packed red cells.

### 2.6 Outcome and Follow‐Up

He was extubated on the third day of admission, after tolerating a nine‐hour T‐piece trial with adequate oxygen saturation, and nebulised adrenaline was continued thereafter.

The facial and airway oedema resolved and his laboratory indices improved steadily. He was discharged home in good clinical condition after 8 days of admission and was booked for outpatient review, including a haemoglobinopathy and iron screen.

## 3 Discussion

Multiple bee stings can result in a broad spectrum of clinical manifestations, ranging from localized reactions to severe systemic toxicity. The severity of envenomation is largely influenced by the number of stings and the cumulative venom burden, with massive envenomation associated with complications such as intravascular haemolysis, rhabdomyolysis, hepatic injury, coagulopathy and acute kidney injury [4, 5, 6].

In our patient, the severity of envenomation was evident from the extensive exposure, with more than 200 stings in a 22 kg child, followed by airway compromise, profound anemia, rhabdomyolysis and marked hepatocellular injury. This combination illustrates that bee venom toxicity is a multisystem process rather than an isolated allergic event [12].

The airway is often the earliest system to be threatened. Facial and oropharyngeal swelling may progress rapidly after stings to the head and neck [13]. In our patient the combination of severe respiratory distress, hoarseness and worsening facial oedema prompted early intubation, and delay in such circumstances may result in a failed airway as swelling progresses.

Severe anemia in massive envenomation results largely from venom‐induced intravascular haemolysis, mediated by melittin and phospholipase A2 [7, 14]. In our patient the hemoglobin of 3.2 g/dL, together with a raised lactate dehydrogenase, reflected significant haemolysis and required urgent transfusion.

Rhabdomyolysis is another recognized feature of massive envenomation [15]. In our patient it was reflected by an elevated creatine kinase with myoglobinuria, and by an AST that was disproportionately elevated relative to ALT. Both haemolysis and rhabdomyolysis release pigments that may precipitate acute kidney injury.

Acute kidney injury following bee stings is multifactorial, arising from pigment nephropathy, hypovolemia, direct venom cytotoxicity and the haemodynamic consequences of anaphylaxis [4, 5, 7, 9, 10]. Although these mechanisms were potentially operative in our patient, renal function remained normal throughout, which we attribute to early presentation, prompt fluid resuscitation and correction of the anemia.

Hepatic injury with marked transaminase elevation, as seen in our patient, has been described in massive envenomation and generally resolves with supportive care [16]. The falling enzyme values in our patient paralleled his clinical recovery.

The microcytic hypochromic indices at presentation suggest a background of iron deficiency or a haemoglobinopathy trait, both common in this setting. Preexisting anemia is likely to worsen the impact of acute haemolysis, and screening at follow‐up is therefore appropriate.

Because systemic toxicity may be delayed by 24 to 72 h, children who have sustained a large number of stings warrant admission and observation, with serial monitoring of hemoglobin, renal function, liver enzymes and muscle enzymes, even when they initially appear well.

### 3.1 Contextual and Resource Considerations

Creatine kinase and lactate dehydrogenase were measured only once during the admission, and haptoglobin, serum tryptase and cardiac markers were not available. These constraints reflect the diagnostic capacity of our setting rather than the clinical course, and they did not prevent recognition and successful treatment of the multisystem toxicity.

## 4 Conclusion

This case highlights that multiple bee stings can lead to severe multisystem toxicity involving the airway, blood, muscle, and liver, and that such children may require intensive care support. Early recognition of the multisystem nature of bee venom toxicity, prompt airway control, and aggressive supportive management can result in full recovery even in settings with limited resources.

## Author Contributions

**Auson Albert:** conceptualization, methodology, writing – original draft, writing – review and editing. **Kelvin M. Musa:** conceptualization, methodology, data curation, writing – original draft, writing – review and editing, resources. **Jane G. Zakaria:** conceptualization, methodology, writing – review and editing, writing – original draft. **Elton Meleki:** conceptualization, supervision, writing – review and editing, writing – original draft, investigation, methodology. **Ronald G. Mbwasi:** writing – review and editing, writing – original draft, supervision. **Anorld David:** writing – review and editing, writing – original draft, conceptualization, methodology, data curation, supervision.

## Funding

The authors have nothing to report.

## Ethics Statement

As a single‐case report with the patient's signed consent, no other ethical review was required.

## Consent

Written informed consent was obtained from the patient's parent/legal guardian for the publication of this case report and the accompanying clinical image.

## Conflicts of Interest

The authors declare no conflicts of interest.

## Data Availability

The data used in this article are available upon request from the authors.
